# Hand Scrubbing and Donning of Sterile Surgical Gloves: An Observational Clinical Audit of Novice Dental Surgeons

**DOI:** 10.7759/cureus.43504

**Published:** 2023-08-15

**Authors:** Nutan Dhamdhere, Rozina Vishnani, Amit Reche, Priyanka Paul, Samruddhi Rathi, Akarsh Bolenwar

**Affiliations:** 1 Public Health Dentistry, Sharad Pawar Dental College, Datta Meghe Institute of Higher Education and Research, Wardha, IND; 2 Oral and Maxillofacial Surgery, Sharad Pawar Dental College, Datta Meghe Institute of Higher Education and Research, Wardha, IND

**Keywords:** oral and maxillofacial surgery, world health organisation, surgical site infections, donning sterile surgical gloves, hand scrubbing

## Abstract

Background

The most critical factors in the satisfactory recovery of a patient post-surgery are obedience to sterilization and aseptic protocol. Using aseptic principles, the standard hand scrubbing and gloving procedure prevents contamination of the surgical site and aids in infection control.

Methods

Eighty dental interns were observed during minor oral surgical procedures for hand scrubbing and donning sterile surgical gloves, following the steps and guidelines provided by World Health Organization (WHO). The dental interns were evaluated, and in order to enhance their understanding of hand scrubbing and donning surgical gloves, desensitization programs were conducted through lectures using PowerPoint presentations. After one week, the participants were observed and evaluated again. This program made the participants aware of asepsis and infection control in clinical practice.

Results

Prior to intervention, only 37.14% of young dental surgeons performed proper conventional hand hygiene practices. After the intervention, this percentage increased to 62.142%, indicating a significant improvement. Regarding the donning of sterile surgical gloves, 43.75% of participants followed the standard steps before the intervention. After the intervention, the percentage raised to 86.25% indicating substantial growth.

Conclusion

Observations before and after the evaluation demonstrated significant changes in the acceptance rates for the fundamental criteria of hand hygiene and donning sterile surgical gloves. Adhering to both procedures according to WHO guidelines will help to reduce the risk of infections and raise awareness about asepsis in the practice among young dental surgeons.

## Introduction

Prevention of surgical site infections (SSI) is one of the perioperative team's top priorities. The term "SSI" refers to an infection that occurs as a result of microorganisms spreading to the patient's wound after an operation [1,2]. It is the most prevalent kind of nosocomial infection in people who have had surgery [1,3]. Infections are the most frequent adverse effects following a hospital stay, affecting 5-10% of hospitalized patients in developed countries. Even though practicing good hand hygiene is a pretty basic habit, only about 40% of healthcare professionals follow it [4-7]. The "Time strokes method" refers to the vigorous scrubbing of the nail beds on the palm resulting in foam [8]. A significant adjustment advocated for hand hygiene practice has been the substitution of waterless alcohol-based substances for conventional handwashing [9,10]. When non-medicated chemicals are replaced with alcohol-based hand wash containing ethanol and isopropyl alcohol, the incidence of contamination declines because the microorganisms present on the operator's hands are killed. Effective monitoring and reporting of hospital-acquired infections (HAIs) is critical for evaluating control mechanisms within healthcare systems and implementing the necessary changes. However, such monitoring can be costly, posing an enormous problem to healthcare systems around the world, particularly in impoverished countries [11].

Putting on gloves before surgery and taking them off during surgery constitutes some of the most common ways for a surgeon to infect oneself and the disinfected operative area. There are two ways to depict donning sterile surgical gloves i.e., open and closed gloving technique. The scrub person's hands should remain inside the sleeves and not touch the cuffs when using the closed-glove technique. The scrubbing person's hands slip all through the sleeves and beyond the cuffs when using the open-glove technique. Previous research has shown that the interface between the glove cuff and the gown is where contamination occurs most frequently [12-14]. Scrubbing, barrier garments, gloving, drapes, and tool sterilization are all essential aseptic measures for maintaining the sterile field's integrity. However, due to the hurried nature of the operating room (OR), the limited amount of training time, and the lack of skilled medical personnel dental students find it tough to develop these skills. As in minor surgical procedures of oral surgery, two techniques are most important while treating patients: hand hygiene and surgical gloving. Before we start a surgical procedure, we should follow all hand hygiene steps according to WHO guidelines [3,15]. WHO stated that instead of concentrating merely on the type of hand hygiene items, healthcare personnel should be encouraged to practice good hand hygiene by concentrating on certain elements that are currently known to have a major impact on behavior. The plan should be diverse, multimodal, and involve implementation support from senior executives and the promotion of education [7,9,16-19]. Following all the steps of gloving is of utmost importance to prevent the spread of infection while doing minor procedures such as exodontia, arch bar placement, third molar surgery, etc. This procedure is important to follow as this will help to prevent post-operative infection in patients. This surgical audit intends to evaluate the procedures like hand scrubbing and donning gloves in young dental surgeons in the Oral and Maxillofacial Surgery Department.

## Materials and methods

After approval from the Institutional Ethical Committee at Datta Meghe Institute of Higher Education and Research with approval number DMIHER(DU)/IEC/2023/1071, this four-week clinical review was conducted at the Department of Oral and Maxillofacial Surgery's minor oral surgery room at Sharad Pawar Dental College, Wardha. The study included 80 young dental surgeons undergoing internship and having adequate knowledge about sterilization and asepsis, and hence the population was selected. To create the methodology, planning, and effects of this research, an in-depth review of the literature was executed.

Pre-evaluation

In the first week, all participants were monitored while scrubbing their hands and donning surgical gloves prior to minor oral surgery procedures. The observation was noted and compared to WHO standards for hand scrubbing and donning sterile surgical gloves. To assess all interns, we took the following evaluation criteria (Tables 1-2). The procedures outlined in the tables assess the effectiveness of young dental surgeons' hand hygiene and donning sterile surgical gloves. In Table 1 for hand scrubbing, every 'yes' signifies one correct step followed and each 'no' indicates one incorrect step from an entire set of ten steps. Similarly in Table 2 for donning sterile surgical gloves from a total of eight steps same criteria were applied. We calculated compliance with these 10 criteria for hand cleaning and eight criteria for donning sterile surgical gloves for each participant and totaled the outcomes of all those involved in each criterion individually. These percentages were mainly utilized to calculate the general compliance of all participants to each criterion.

The sensitization

**Table 1:** Pre-evaluation and Post-evaluation Compliance Percentage Improvement for Various Steps in Hand Scrubbing

	Adherence % rates		
Criteria	Pre-evaluation	Post-evaluation	% Improvement
1. Whether nails are trimmed?	60	80	20
2. Whether the hand is free of ornaments and accessories till the elbow?	50	80	30
3. Is his/her hand moist up to the elbow and does he/she use sufficient soap water to obtain an adequate lather?	40	70	30
4. Whether they wipe both thenars simultaneously?	80	100	20
5. Whether hands are rubbed back and forth.	60	90	30
6. Whether or not they have interlinked his or her fingers.	50	70	20
7. Whether they have Cup his/her Fingers?	50	100	50
8. Whether they have cleaned the Thumbs?	40	80	40
9. Whether they scrub their palms with his/her digits?	30	100	70
10. Whether they have rinsed his/her hands properly to the elbow.	60	100	40
Mean	37.14±12.23	62.142 ±20.45	30.002 ±16.34

**Table 2:** Pre-evaluation and Post-evaluation Compliance Percentage Improvement for Various Steps in Donning Sterile Surgical Gloves

	Adherence rates %		
Criteria	Pre-evaluation	Post-evaluation	% Improvement
1. Keep hand above waist without any contact with the non-sterile area	60	100	40
2. Choose the proper glove size	50	90	40
3. Open the outer package without touching it to the sterile surface and place the inner packet on the sterile surface	40	70	30
4. Perform the hand hygiene procedure	60	100	40
5. Open the inner packet and grip one glove's rolled cuff edge with one hand’s thumb and index finger.	40	80	40
6. Slip the other hand inside the glove in one quick motion, keeping the rolled cuff at wrist level.	30	70	40
7. Grab up another glove by placing your gloved hands' digits beneath the cuff of the glove.	20	80	60
8. Maintain contact with a sterile area	50	100	50
Mean	43.75 ±20.33	86.25 ± 37.41	42.5 ±19.93

Following the first week of gathering information and observation, everyone who participated was given a presentation. It comprises a demo video of the conventional hand scrubbing and surgical gloving approach according to WHO recommendations. Subsequently, third-year residents of the oral surgery department also gave an individual demonstration of performing each and every step of hand scrubbing and gloving in accordance with WHO guidelines in the minor oral surgical room to the participants. Subsequently, third-year residents of the oral surgery department also gave an individual demonstration in the minor oral surgical room to the participants. A pictorial guide for hand scrubbing was also displayed inside the minor oral surgical room.

Post-evaluation

After one week of intervention, all participants were inspected again for compliance with the hand scrubbing and standard surgical gloving criteria in the subsequent phase of the audit, i.e., the final week. Each correctly completed step received one mark, and all 10 stages for hand scrubbing and eight steps for donning sterile surgical gloves were examined free of constraints. Everyone was presented with a total score, and the average improvement was computed by putting all the compliance scores together. We calculated compliance with these 10 criteria for hand scrubbing and eight criteria for donning sterile surgical gloves for each participant and added the scores of all participants to each criterion independently. The overall conformance of all participants to each criterion was calculated using percentages.

## Results

**Figure 1:**
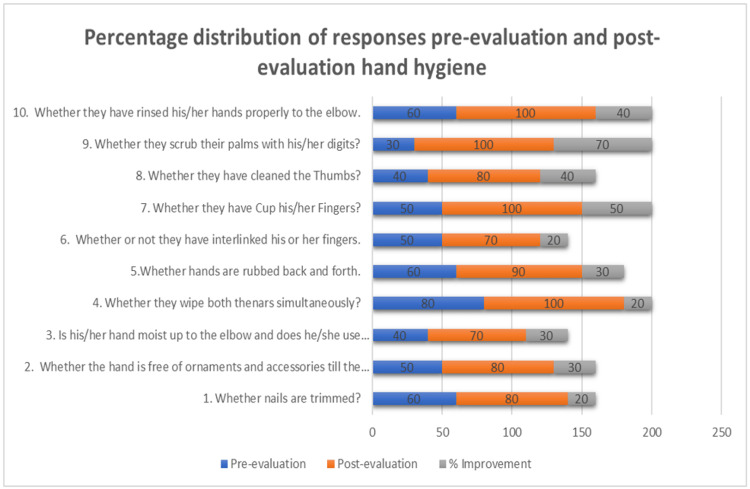
Pre-evaluation and Post-evaluation Compliance Percentage Improvement for Various Steps in Hand Scrubbing

**Figure 2:**
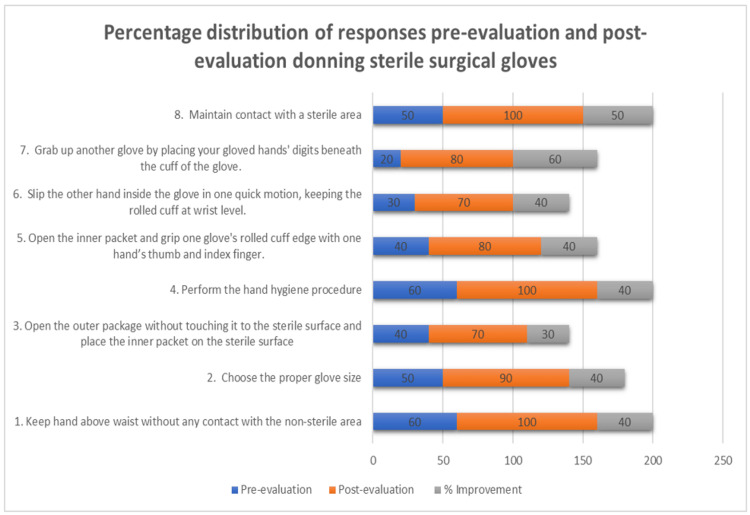
Pre-evaluation Post-evaluation Compliance Percentage Improvement for Various Steps in Donning Sterile Surgical Gloves

Eighty young dental surgeons, 55 females and 25 males, were observed for the purpose of awareness and implementation of conventional hand hygiene skills, as well as their use of sterile surgical gloves. The cumulative percentages of compliance of all participants to each criterion separately for hand hygiene had the smallest difference of 20% because these processes were precisely followed by all participants. In addition, 30% of candidates disregarded criterion 3 for donning sterile surgical gloves: opening the outer package without touching it to the sterile surface and placing the inside packet on the sterile surface. The greatest rate of growth was shown in criteria 9, where 70% of participants gained knowledge to scrub their palms with their digits, with a 70% variance among pre-post-intervention. In the instance of hand hygiene, all participants accomplished 37.14% of the standard guidelines during pre-sensitized observation, however, 62.142% met them afterward the sensitization. The percentage of rise in before and after intervention, hand hygiene compliance rates remained exceptional at 30.002%. In the case of wearing sterile surgical gloves, interns met 43.75% of the demands during the pre-intervention inspection and 86.25% after the sensitization. The rate of increase in surgical glove compliance rates is impressive at 86.25%. After taking the feedback from the participants about the sensitization the maximum preferred was the individual demonstration as shown in (Table 3). The result can be well depicted in the form of a bar chart given in (Figures 1-2). The bar chart displays pre-evaluation in blue color, post-evaluation in orange color and percentage improvement in grey color.

**Table 3:** Best Sensitization Method According to Young Dental Surgeons

	Individual demonstration	Video demonstration	Pictorial guide
Percentage %	77.5	15	7.5

## Discussion

As in minor surgical procedures of oral surgery, while doing patients, two techniques are most important: hand hygiene and surgical gloving. Before we start a surgical procedure, we should follow all hand hygiene steps according to WHO guidelines. If bacteria and viruses enter the bloodstream through patients, it may cause serious infections like hepatitis, HIV, severe acute respiratory syndrome, etc. Following the aseptic technique is of utmost importance to prevent the spread of infection while doing minor procedures such as Exodontia, Arch bar placement, 3rd Molar surgery, etc. This surgical audit intends to evaluate the procedures like hand scrubbing and donning gloves in young dental surgeons in the Oral and Maxillofacial Surgery Department. In our study, the participants fulfilled Criteria 1, 4, and 6 in Table 1 and Criteria 3 in Table 2. Before the intervention, the compliance rate at Lahore General Hospital was 70% and 55%, accordingly [1]. According to a research report released in Nepal in 2022, the average compliance for criteria 4 and 5 is 89% [3].

The most progress was shown by Criteria 9 in Table 1 and Criteria 7 in Table 2 which rose from 30% to 100% and 20% to 80%, respectively. Our participants showed better compliance rates by 100% in criteria 4, 7, 9, and 10 in Table 1 and criteria 4 and 8 in Table 2 respectively. However, in the study published by Biddhya and Bista, which was done in an operating room of an educational medical facility, the compliance rate reached 74 [3]. According to 46 research conducted by various specialists all over the globe, hand hygiene (HH) compliance enhanced after the course of action, ranging from 1% to 66%, with the mean net change being a 26% rise [11]. Hand hygiene and the use of sterile surgical gloves improved by 30.002% and 42.5%, respectively, in the study we conducted. In pre-evaluation, the study by Biddhya and Bista had a mean compliance of 88.88%, but our investigation had a mean of 37.14% and 43.75%, respectively [3]. After observing this, we educated the interns with a pictorial guide, video demonstration, and individual demonstration to explain the steps according to WHO guidelines. After that, we performed a post-evaluation, which revealed that the average compliance was 62.14% and 86.25%, respectively.

According to a study on hand washing and gowning compliance conducted in Indonesia, 83.12% of surgical residents adhered to the rules [20]. In the same study, taking off the gloves' covering by hand from beneath the sleeves of the garment had the lowest mean score, while in our analysis, criteria 9 in Table 1 and criteria 7 in Table 2 had the lowest mean. New research on the in vivo efficacy of alcohol-based hand scrubbing, as well as the low risk of rashes related to their use, is highlighted [10].

An observational study done by Anargh et al. assessed the knowledge about hand hygiene among 100 healthcare workers in tertiary healthcare facilities. The study was questionnaire-based and concluded that inadequate compliance was seen despite training, and alcohol-based rubs provided a false sense of security [21]. A similar study conducted in Nigeria on 173 healthcare workers which included hospital auxiliary staff showed good compliance with hand hygiene practices [22]. After observing all the studies and the present survey conducted by our study, we realized the need to re-audit frequently and sensitize pictorial guides, video demonstrations and individual demonstrations to impart knowledge and improve compliance among healthcare workers.

Limitations of the study

The small sample size is the major limitation of this study. Such studies should be done at many centres for validity. Lack of sufficient review of the literature and original studies on aseptic procedures are other limitations.

## Conclusions

This clinical audit was conducted for the first time in dental college, proving that additional audits must be conducted to improve patient safety. This study investigated how young dental surgeons knew before and after being evaluated for using the WHO-recommended standard sterile surgical glove technique. This study identified significant changes in the variables both before and after the intervention. Despite many interns' greatest attempts to follow the WHO recommendations for hand hygiene and surgical gloving methods, many still need to live up to expectations. On the contrary, as this study shows, the training sessions resulted in quite a lot of progress. Therefore, we must regularly undertake clinical audits and resensitize operating personnel to make optimistic changes to clinical practice. Based on the previous data, more regular audits should be performed to increase adherence to accepted protocols. This can result in a major improvement in eliminating health risks, which can still aid a nation's economy by lowering the number of prolonged hospital stays and the burden of iatrogenic diseases.
